# Supplementary material for: Atomically-precise dopant-controlled single cluster catalysis for electrochemical nitrogen reduction
Source: Nat Commun. 2020 Sep 1;11:4389. doi: 10.1038/s41467-020-18080-w (PMC7463028; doi:10.1038/s41467-020-18080-w)
Supplement: Supplementary file 1 — Supplementary Information [file 41467_2020_18080_MOESM1_ESM.pdf]

Supplementary Information for

Atomically-Precise Dopant-Controlled Single Cluster Catalysis for Electrochemical Nitrogen Reduction

Yao et al.

Supplementary Figures

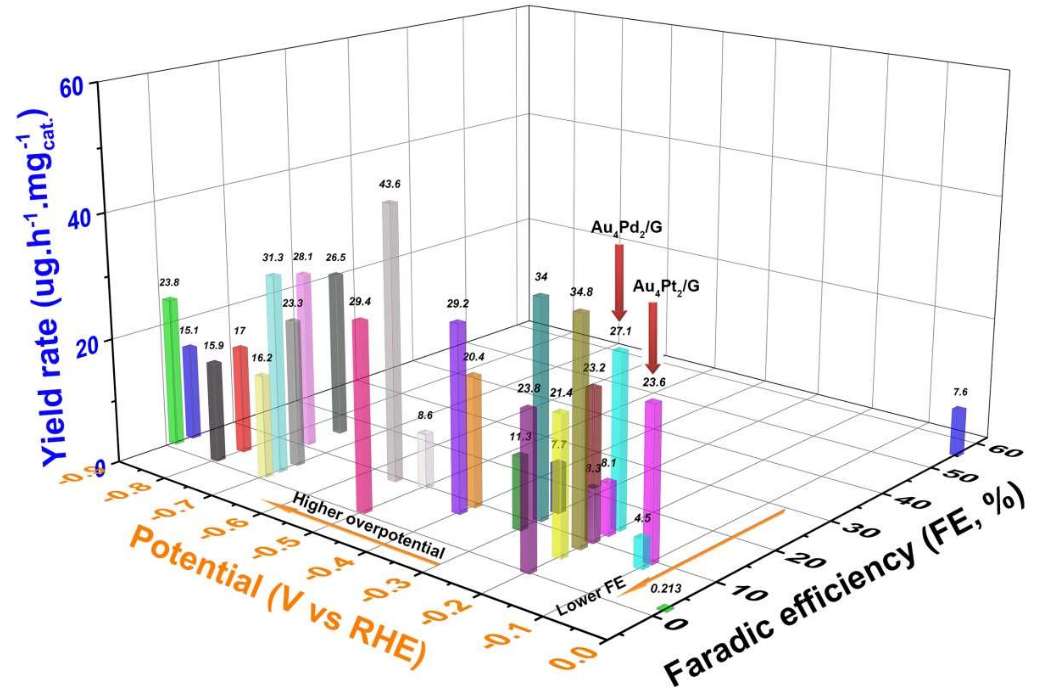

Supplementary Fig. 1 Electrochemical N_2 reduction reactions (ENRR) catalytic performance of the Au_4Pd_2/G and Au_4Pt_2/G catalysts in comparison with the catalysts reported.

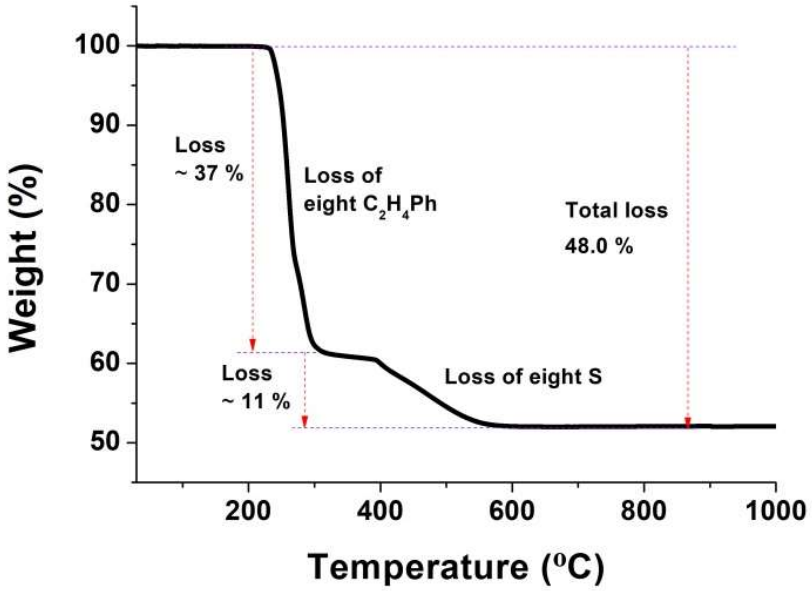

Supplementary Fig. 2 Thermogravimetric analysis (TGA) result of $Au_4Pt_2(SR)_8$ crystals.

The first weight loss of (~37%) can be attributed to the removal of eight -C_2H_4Ph organic part from thoil ligand (-S-C_2H_4Ph), and the subsequent weight loss (~11%) stems from the desorption of eight sulphur atoms of metal cluster. A complete loss of 8 of -SC_2H_4Ph during TGA measurement results in a total weight loss of 48% in the first two steps. Mass spectroscopy (MS) and TGA are two commonly-used characterization techniques for metal clusters. High resolution MS provides the accurate analysis of molecular mass of molecular-like clusters. TGA offers additional information related to the mass contribution of ligand involved in the cluster. The molecular weight of as-prepared $Au_4Pt_2(SC_2H_4Ph)_8$ cluster is determined to be 2274 Da (the peak with the largest M/Z value as shown in Figure 1a). The mass contribution of ligand is determined to be 48% based on the TGA result. Therefore, the mass of ligand can be calculated as 2274 × 48% = 1091.5 Da. The total number of ligand can be calculated as 1091.5/137(the molecular weight of SC_2H_4Ph) = 7.97, in consistent with the total eight SC_2H_4Ph ligands for each cluster (Supplementary Fig. 2). Based on the aforementioned analysis, the molecular composition of the cluster can be deduced as $Au_4Pt_2(SC_2H_4Ph)_8$, which is further corroborated by the theoretical isotopic MS patterns of $Au_4Pt_2(SC_2H_4Ph)_8$ (the inset of Fig. 1a).

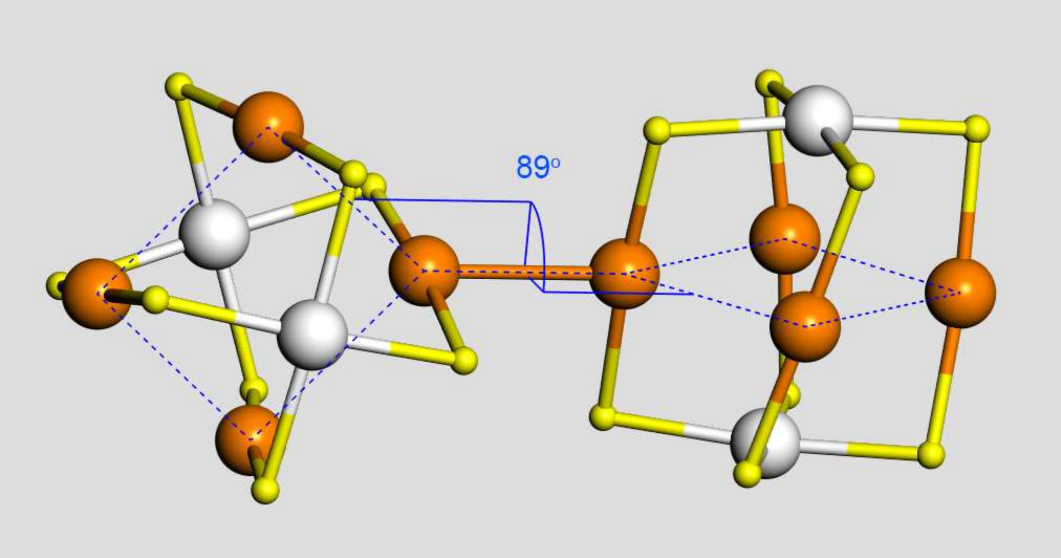

Supplementary Fig. 3 One-dimensional (1D) polymeric $Au_4Pt_2(SR)_8$ chain. White, orange, yellow balls represent the platinum, gold, sulfur atoms. C and H atoms are omitted for clarity.

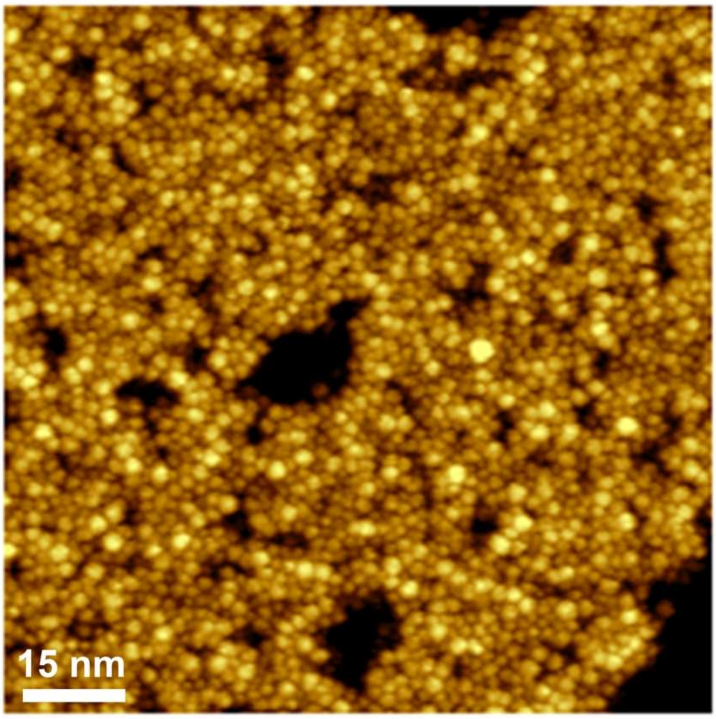

Supplementary Fig. 4 STM image of $Au_4Pt_2(SR)_8$ clusters on HOPG upon annealing at 100 °C.

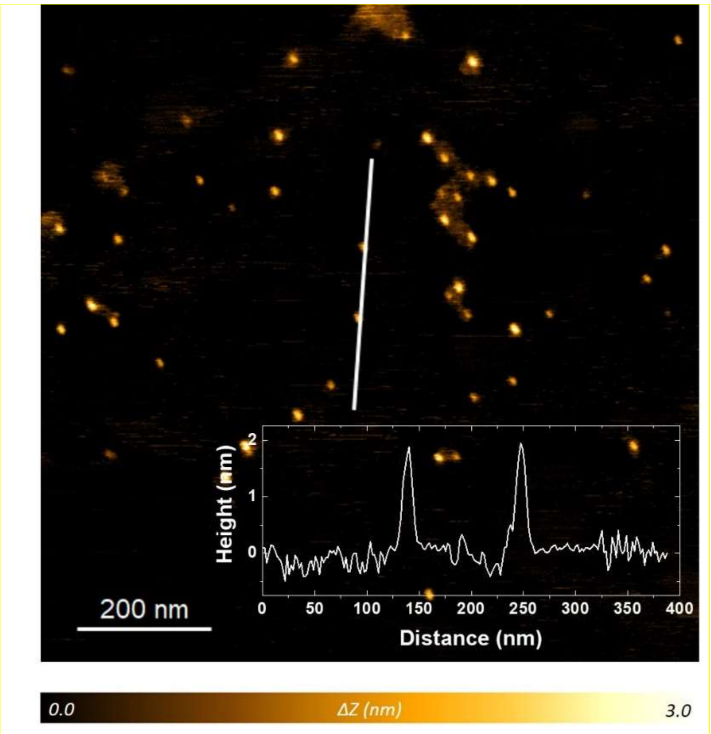

Supplementary Fig. 5 AFM images of $Au_4Pt_2(SR)_8$ clusters deposited on HOPG. The inset represents an AFM height profile acquired along the dotted white line.

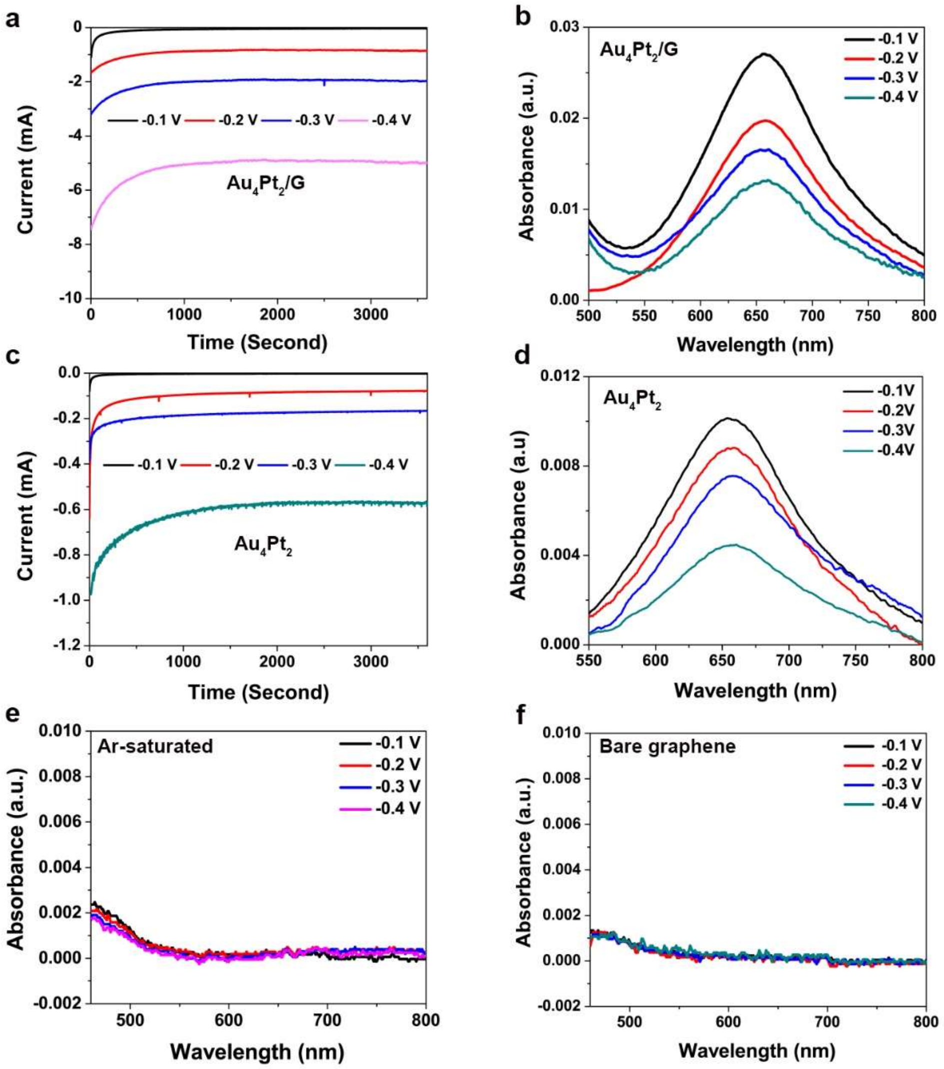

Supplementary Fig. 6 Electrochemical N_2 reduction using Au_4Pt_2/G and Au_4Pt_2 catalysts, respectively. (**a**) Chrono-amperometry profiles at each applied potential using Au_4Pt_2/G catalyst. (**b**) UV-Vis absorption spectra of the electrolyte after performing ENRR for 1 hour at different applied potentials using Au_4Pt_2/G catalyst (indophenol method). (**c**) Chrono-amperometry profiles at each applied potentials using unsupported Au_4Pt_2 catalyst. (**d**) UV-Vis absorption spectra of the electrolyte after performing ENRR for 1 hour at different applied potentials using unsupported Au_4Pt_2 catalyst (indophenol method). (**e**) Control experiments conducted in an Ar-saturated electrolyte. (**f**)

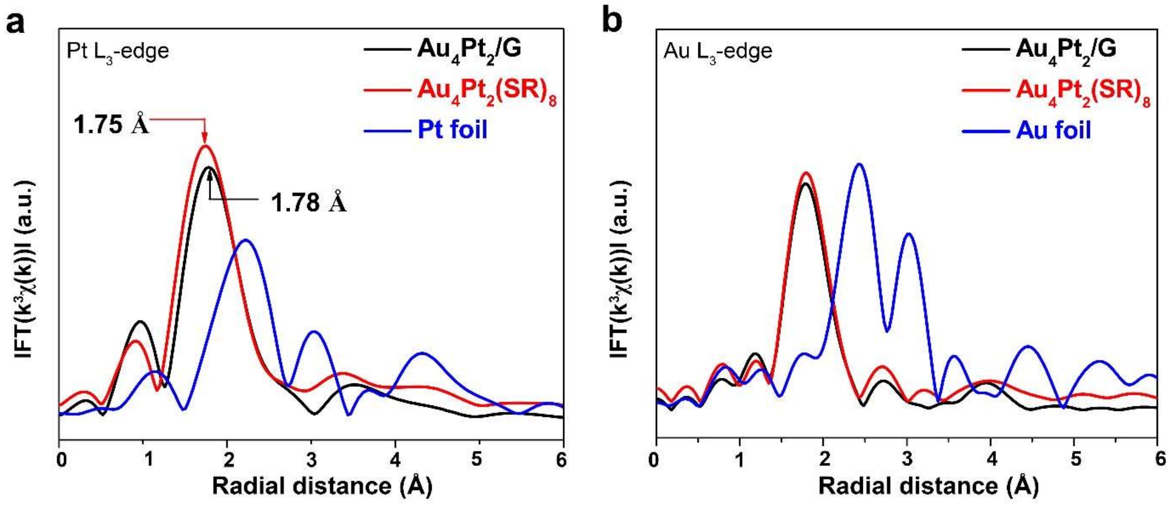

Supplementary Fig. 7 The Fourier transform of the K3-weighted EXAFS spectra taken for the different samples: Au_4Pt_2/G (black curve), $Au_4Pt_2(SR)_8$ (red curve) and Pt foil (blue curve). (**a**) Pt L_3-edge. (**b**) Au L_3-edge.

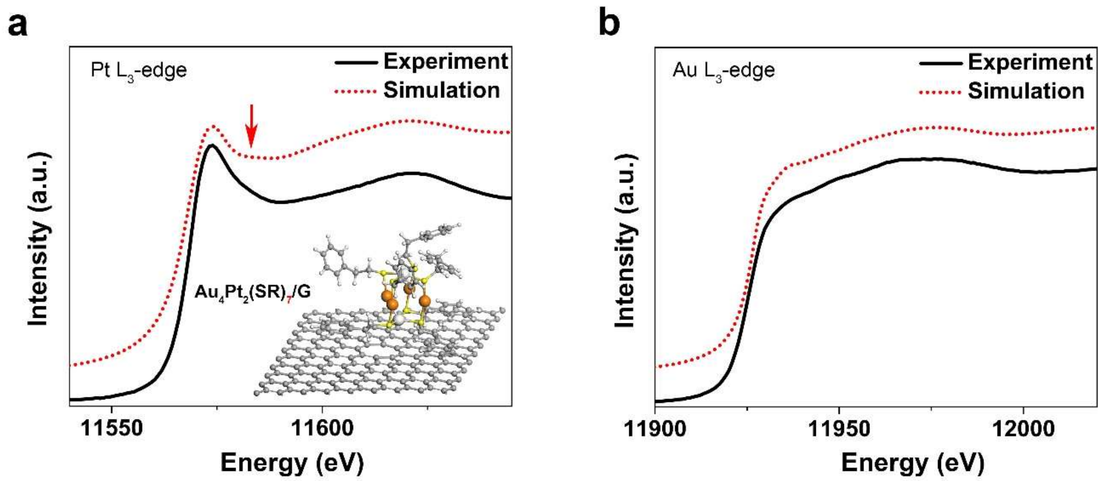

Supplementary Fig. 8 (a) Pt L_3- and (b) Au L_3-edges XANES spectra. Inset of **a**: DFT-optimized structure: $Au_4Pt_2(SR)_7$ adsorbed on graphene vacancy. Black curve refers to the experimental data; red dotted line represents the simulated XANES spectra of the DFT-optimized structure, $Au_4Pt_2(SR)_7$/G (inset).

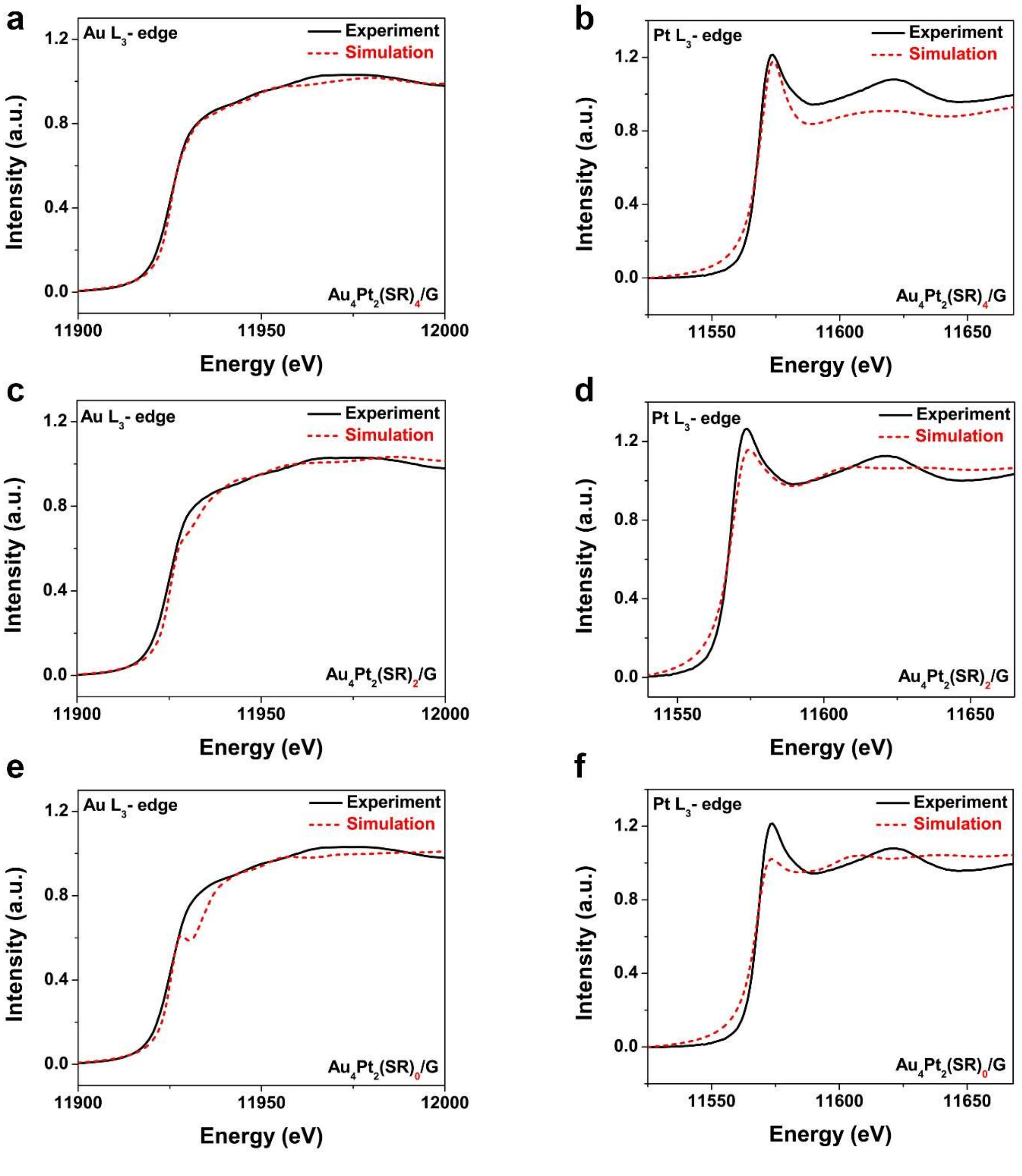

Supplementary Fig. 9 A comparison of experimental XANES spectra with the simulated spectra of $Au_4Pt_2(SR)_4$/G, $Au_4Pt_2(SR)_2$/G and $Au_4Pt_2(SR)_0$/G. (**a,b**) L_3-edges of Au and Pt for $Au_4Pt_2(SR)_4$/G; (**c,d**) L_3-edges of Au and Pt for $Au_4Pt_2(SR)_2$/G; (**e,f**) L_3-edges of Au and Pt for $Au_4Pt_2(SR)_0$/G.

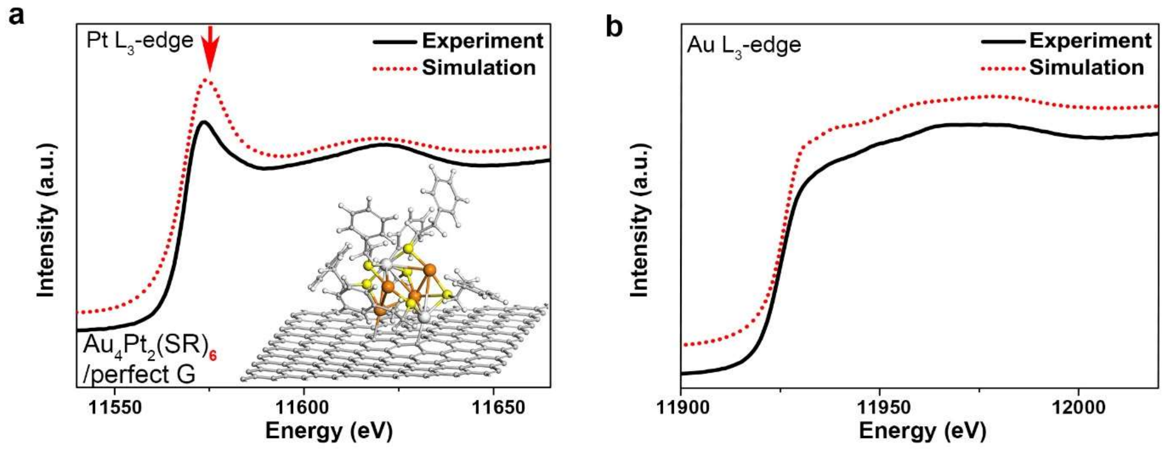

Supplementary Fig. 10 (a) Pt L_3- and (b) Au L_3-edges XANES spectra. Inset of **a**: DFT-optimized structure: $Au_4Pt_2(SR)_6$ adsorbed on perfect graphene. Black curve refers to the experimental data; red dotted line represents the simulated XANES spectra of the DFT-optimized structure, $Au_4Pt_2(SR)_6$/perfect G (inset).

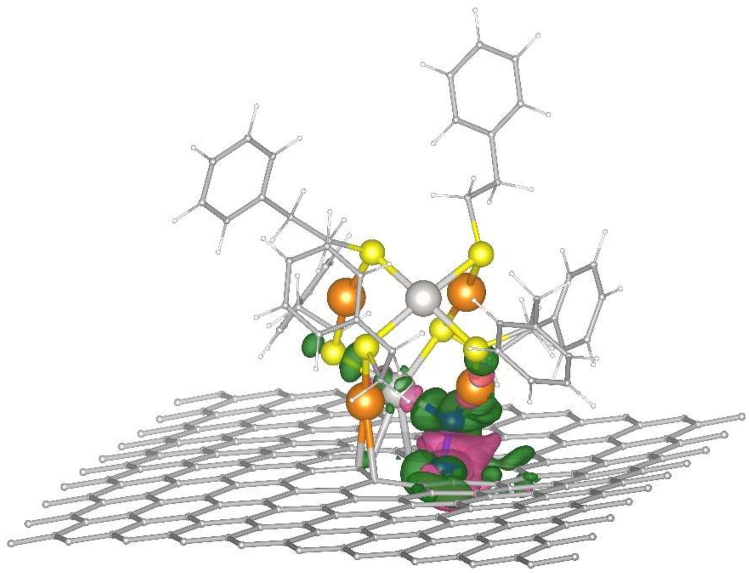

Supplementary Fig. 11 The most stable N_2 adsorption configuration obtained through DFT calculation (with D2 correction).

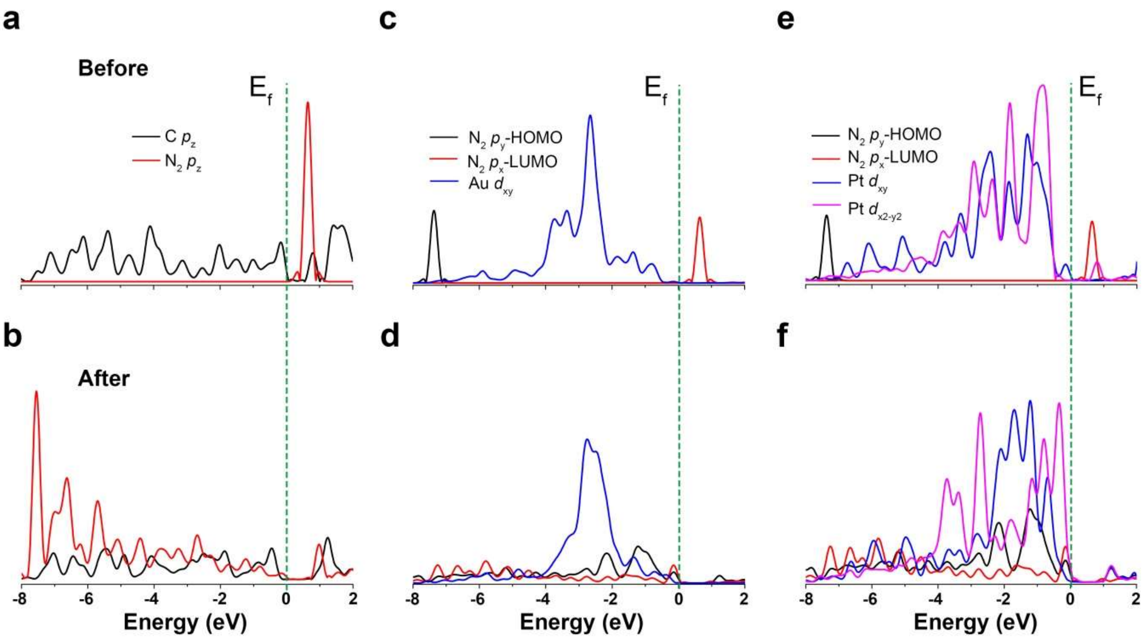

Supplementary Fig. 12 The PDOS of different orbitals. (**a,c,e**) PDOS of different oribtals for non-hybrided structure (isolated N_2 and Au_4Pt_2/G cluser). (**b,d,f**) PDOS of the different orbtials in the hybrided structures (N_2 adsorbed over the catalyst).

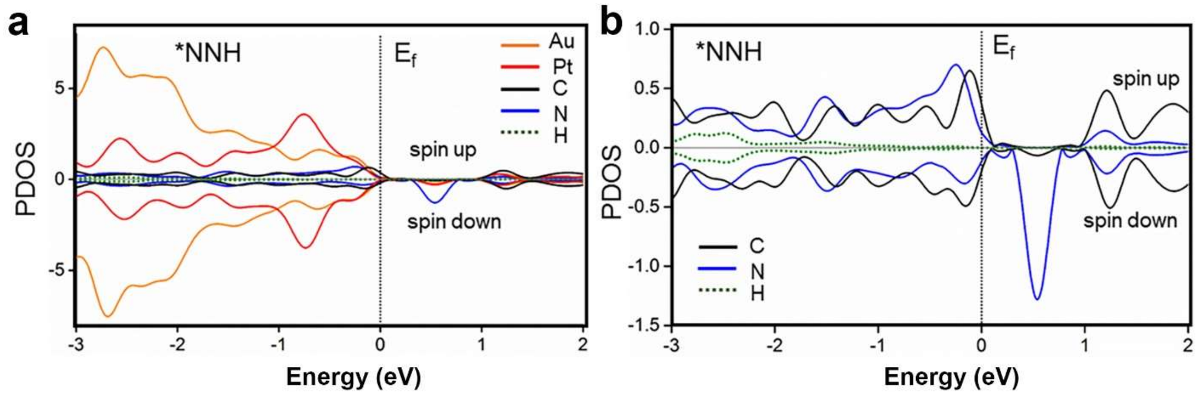

Supplementary Fig. 13 Calculated PDOS of *NNH produced for the pathway under distal mechanism: (**a**) PDOS for all chemical species and (**b**) Enlarged PDOS for N, C and H that are bonded with N_2. It reveals a significant hybridizations between N and both C and H that are bonded with N_2, indicating that N_2 forms chemical bond with both C and H. For a better visualization, the PDOS of H is enhanced by 10 times.

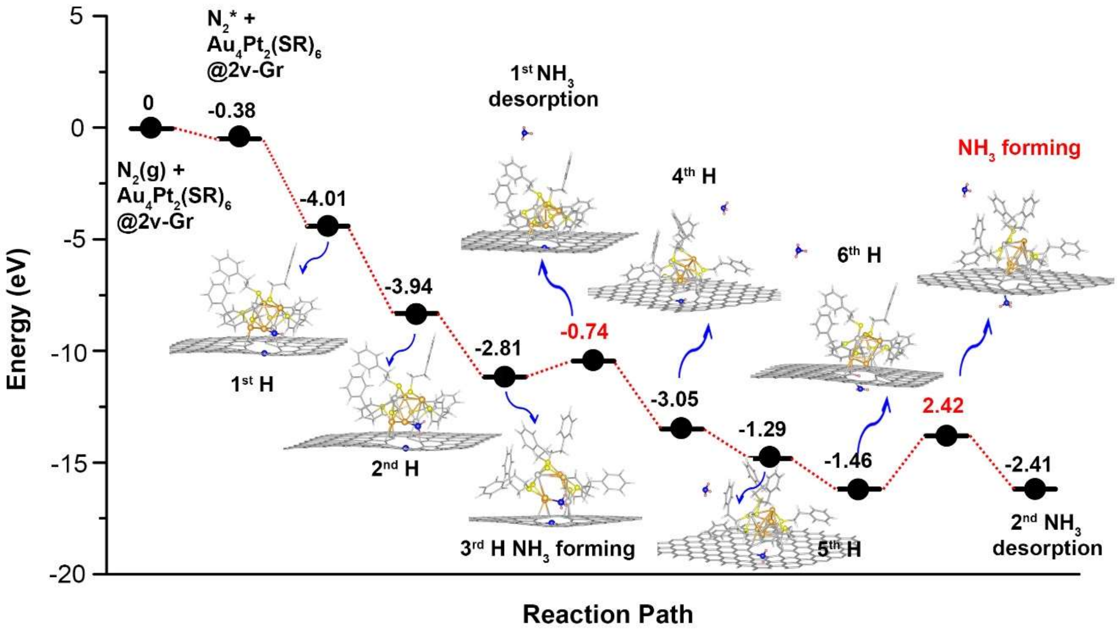

Supplementary Fig. 14 Calculated energy profile of the reaction pathway for the alternative mechanism (pathway II). We present the full reaction path in which the N atom binding with the metal cluster leaves first. For this mechanism, there are two rate-limiting steps, one is the desorption of the first NH_3 with a barrier of 0.74 eV, the other one is the formation of the second NH_3 with a high barrier of 2.42 eV.

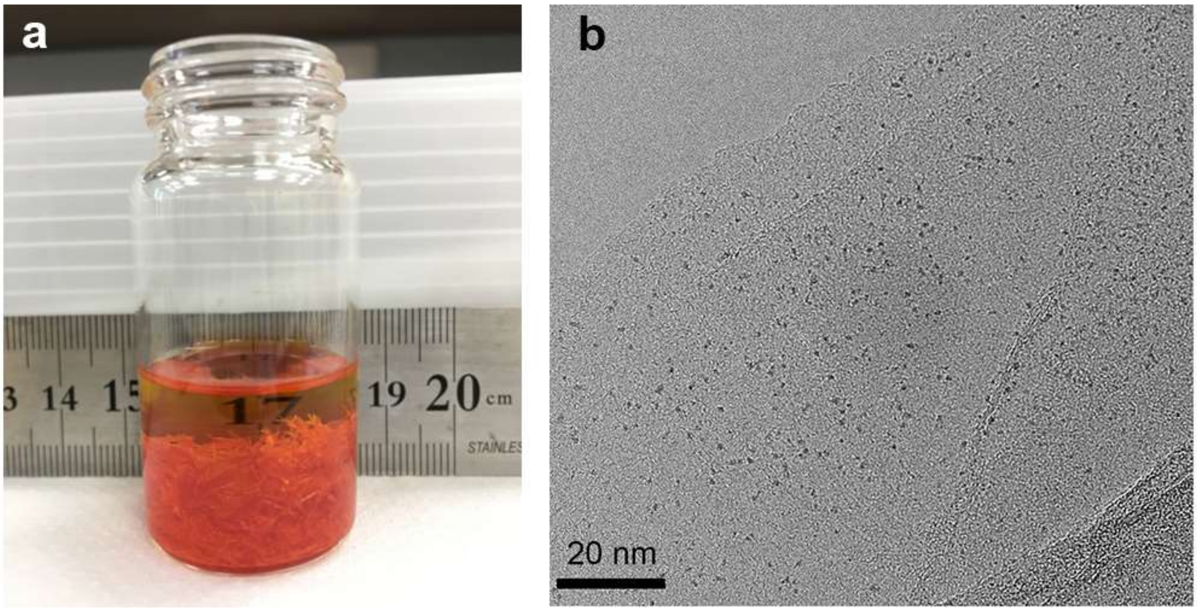

Supplementary Fig. 15 Large-scale synthesis of $Au_4Pd_2(SR)_8$ cluster for the fabrication of Au_4Pd_2/G SCC. (**a**) Photograph of as-obtained $Au_4Pd_2(SR)_8$ crystals. (**b**) TEM image of Au_4Pd_2/G catalyst.

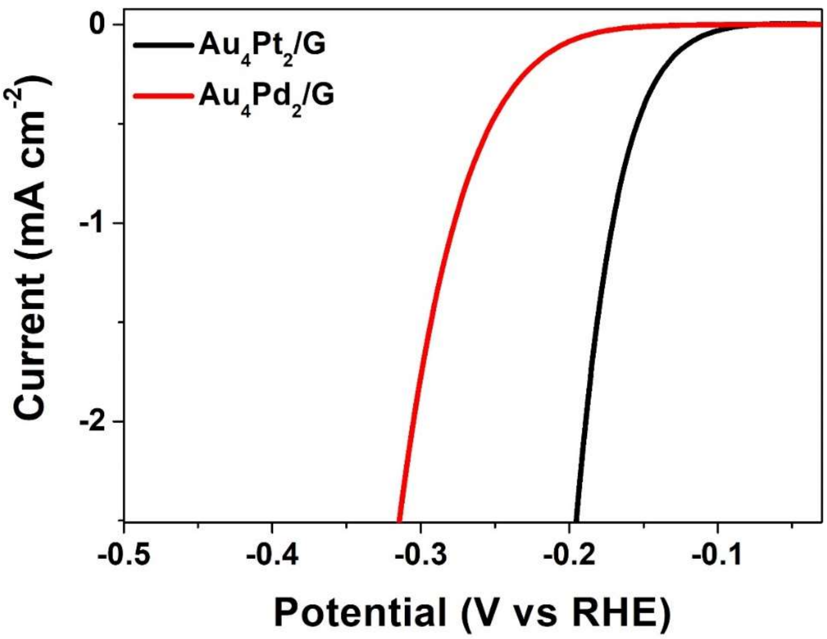

Supplementary Fig. 16 HER performance of Au_4Pt_2/G and Au_4Pd_2/G catalysts. The onset potential of HER is determined to be ~ -0.1 V and -0.2 V for Au_4Pt_2/G and Au_4Pd_2G respectively.

Comparing the ENRR performance of Au_4Pd_2/G and Au_4Pt_2/G, one can find out that the Au_4Pd_2/G catalyst yields a NH_3 production rate of 13.1 μg mg^{-1} h^{-1} at -0.1 V, lower than that of Au_4Pt_2/G catalyst at the same potential. This indicates that Au_4Pd_2/G has a lower ENRR activity compared to Au_4Pt_2/G. However, we obtained a maximum NH_3 yield rate of 27.1 μg mg^{-1} h^{-1} with a FE of ~12% at a more negative potential of -0.2 V for Au_4Pd_2/G, actually outperforming the Au_4Pt_2/G (Fig. 3) at a more negative potential. These observations suggest that hydrogen evolution reactions (HER) could be more effectively suppressed in this system as compared to that of Au_4Pt_2/G. This is also consistent with the fact that Pt generally favors the HER, which severely limits ENRR towards NH_3 production at more negative potentials. To verify this, we have evaluated the HER performance of both Au_4Pd_2/G and Au_4Pt_2/G catalysts. The results clearly demonstrate that the overpotential of Au_4Pd_2/G for HER is higher (more negative) than that of Au_4Pt_2/G, suggesting the competing HER reaction has been more effectively suppressed in the process of ENRR.

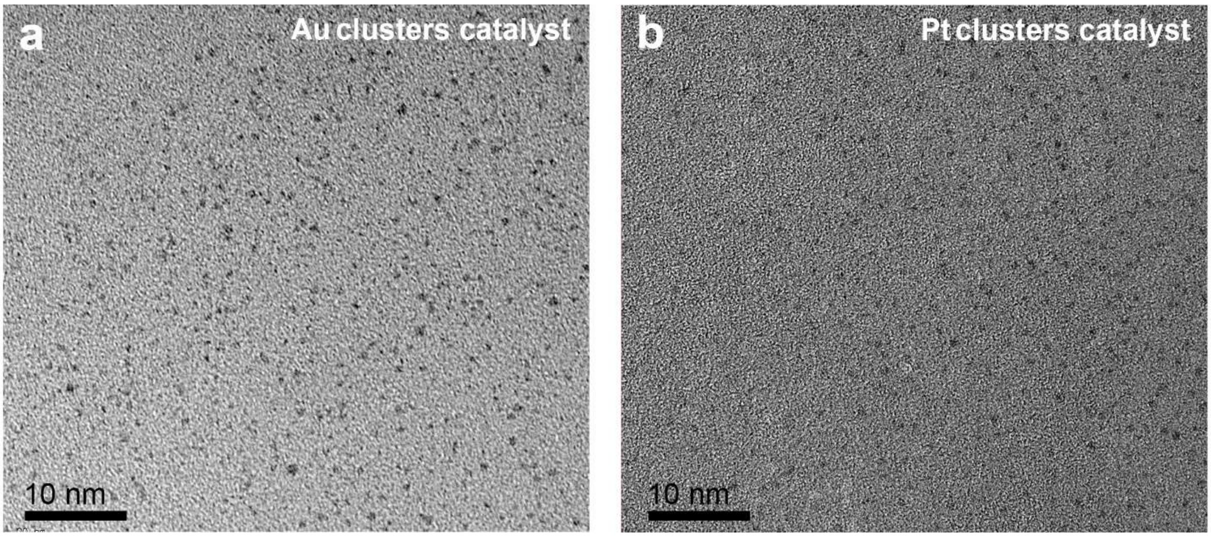

Supplementary Fig. 17 Large area TEM images of pure Au and Pt cluster catalysts. (**a**) TEM image of Au clusters catalyst and (**b**) TEM image of Pt clusters catalyst. (note that both Au and Pt clusters are loaded on the defective graphene)

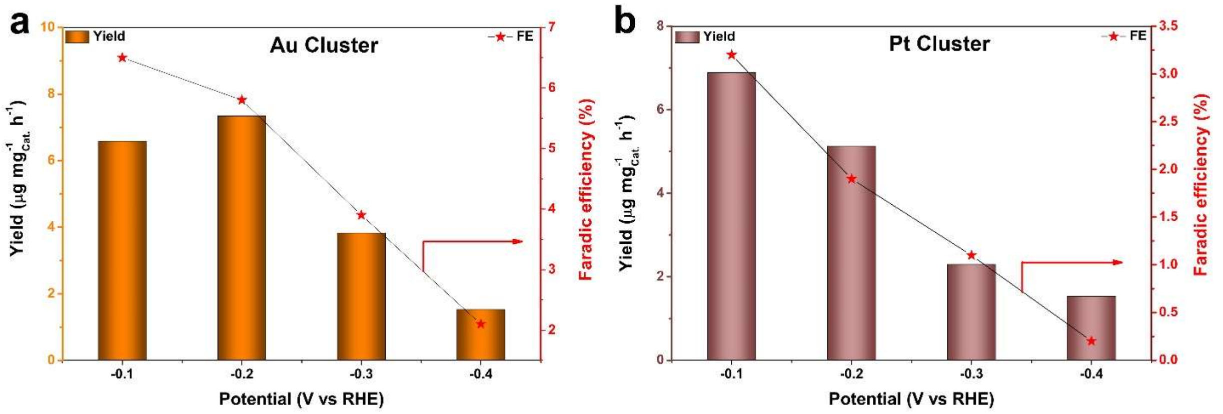

Supplementary Fig. 18 Catalytic performance of pure Au and Pt clusters catalysts in ENRR. (**a**) NH_3 yield rate and FE for Au cluster catalyst and (**b**) NH_3 yield rate and FE for Pt cluster catalyst.

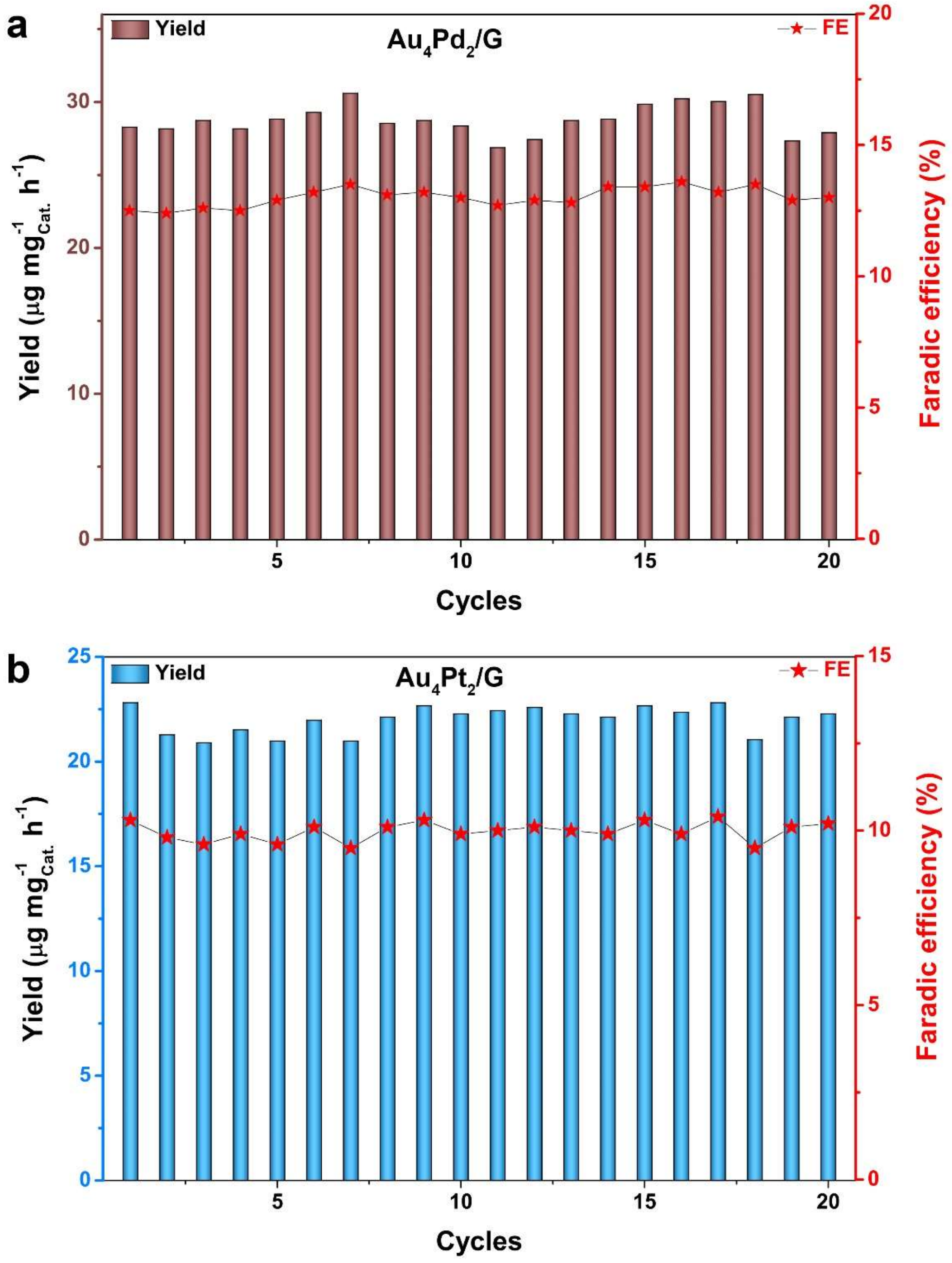

Supplementary Fig. 19 Multiple cycling stability of both Au_4Pd_2/G and Au_4Pt_2/G catalysts for ENRR. (**a**) Au_4Pd_2/G and (**b**) Au_4Pt_2/G.

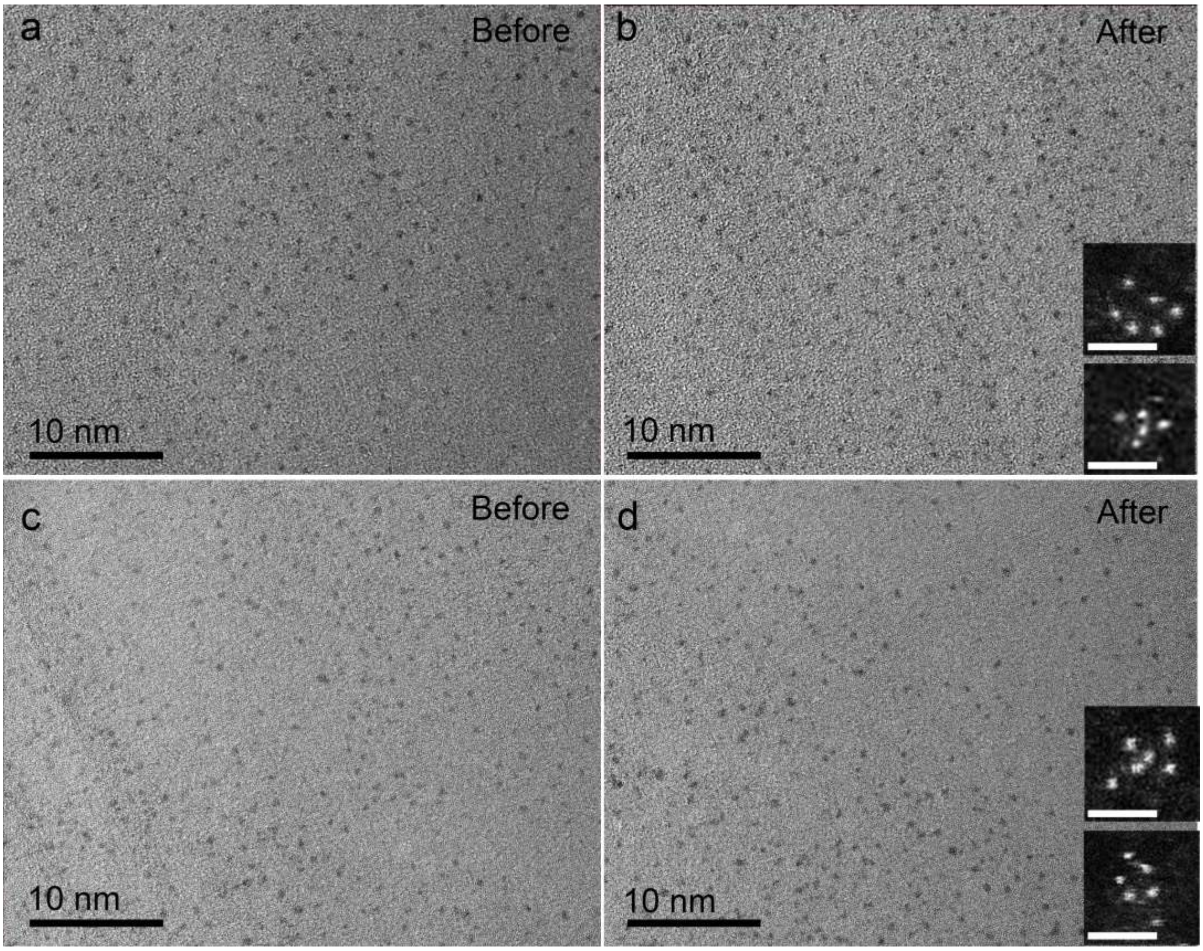

Supplementary Fig. 20 Structural characterization of bimetallic cluster before and after electrochemical N_2 reduction. a, b TEM images of Au_4Pt_2/G before and after ENRR. c, d TEM images of Au_4Pd_2/G before and after ENRR. (Inset b and d are spherical aberration corrected STEM images of Au_4Pt_2/G and Au_4Pd_2/G catalysts after ENRR. Scale bars for inset are 5Å)

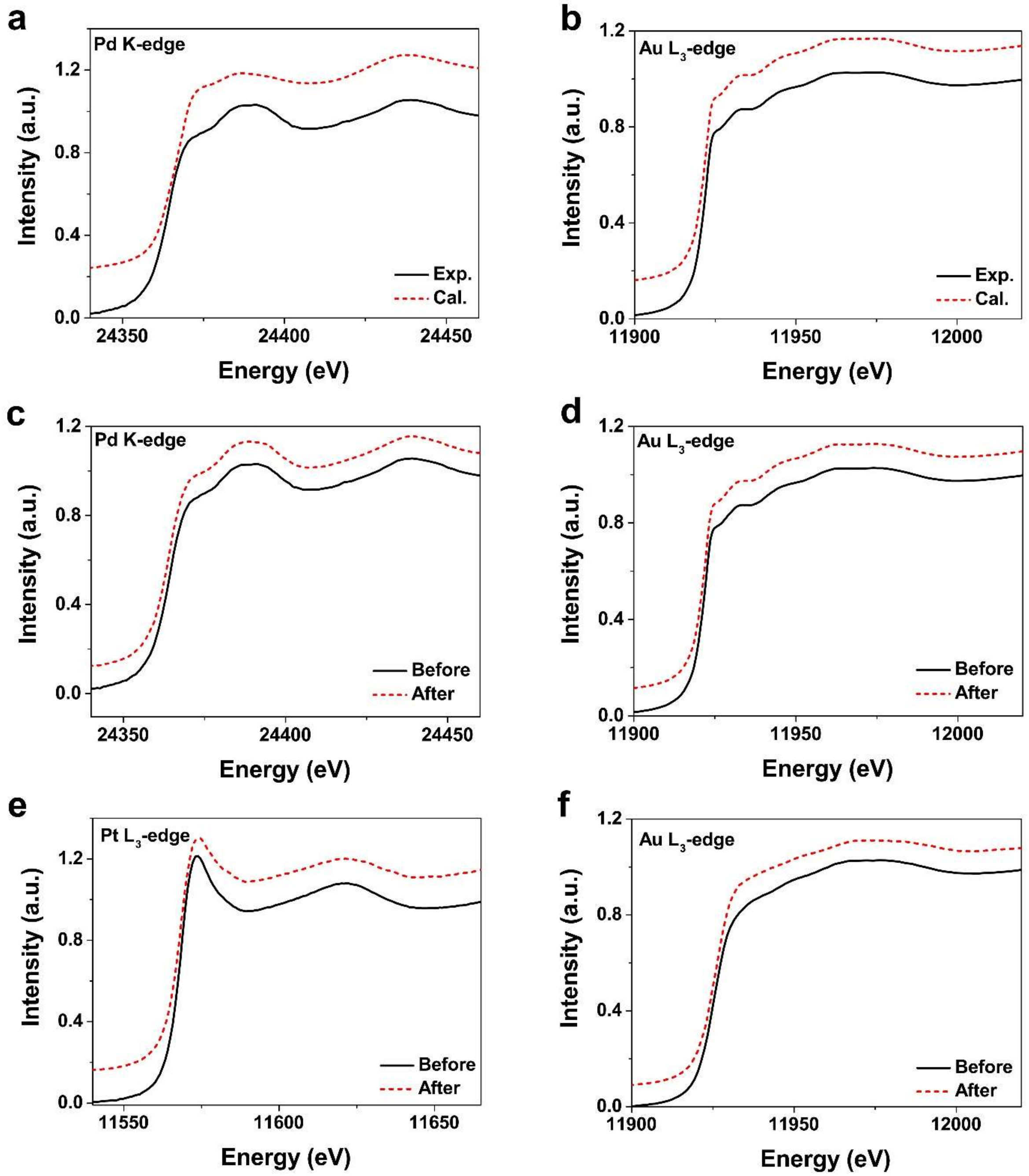

Supplementary Fig. 21 XANES spectra of Au_4Pd_2/G and Au_4Pt_2/G catalysts before and after ENRR. (**a**) Pd K- and (**b**) Au L_3-edges XANES spectra of Au_4Pd_2/G catalyst, (**c**) Pd K-edge and (**d**) Au L_3-edge XANES spectra of Au_4Pd_2/G catalyst before and after ENRR, (**e**) Pt K-edge and (**f**) Au L_3-edge XANES spectra of Au_4Pt_2/G catalyst before and after ENRR.

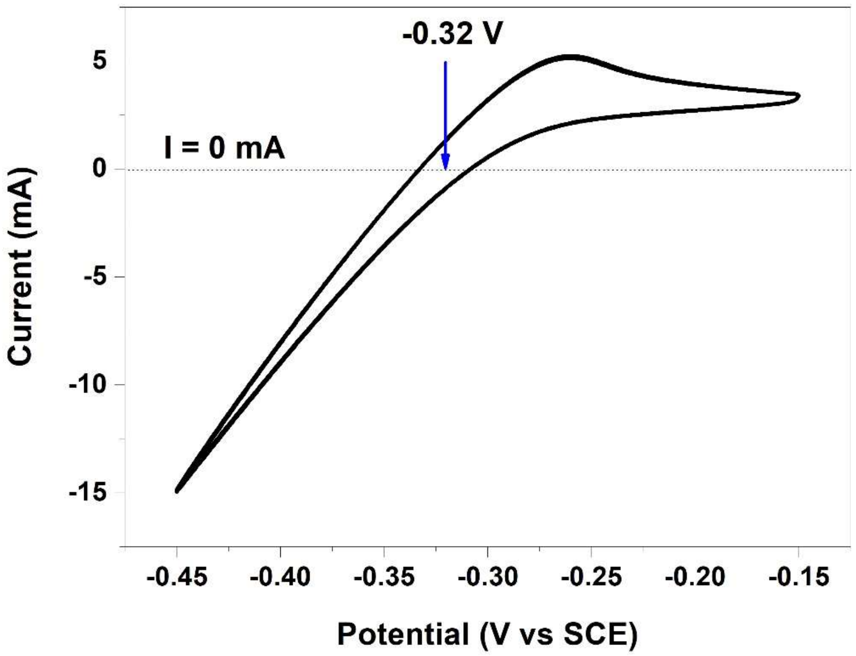

Supplementary Fig. 22 The calibration of SCE for the conversion of electrical potentials with respect to the reversible hydrogen electrode (RHE). E (RHE) = E (SCE) + 0.32V.

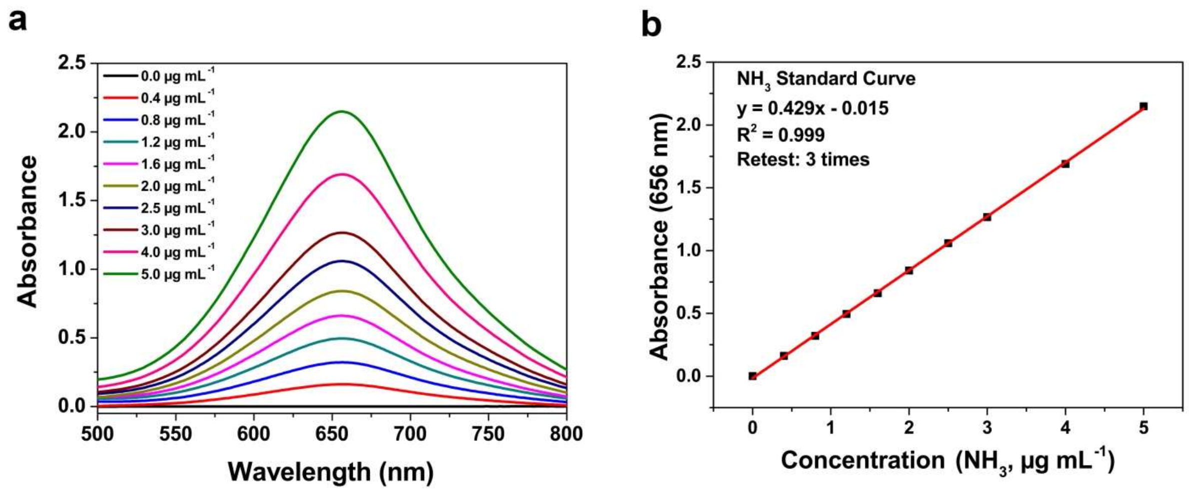

Supplementary Fig. 23 Absolute calibration of the indophenol blue method was achieved using ammonium chloride solutions of known concentration as standards. (**a**) UV-vis curves and (**b**) concentration-absorbance curve of NH_4^+ ions solution with a series of standard concentration.

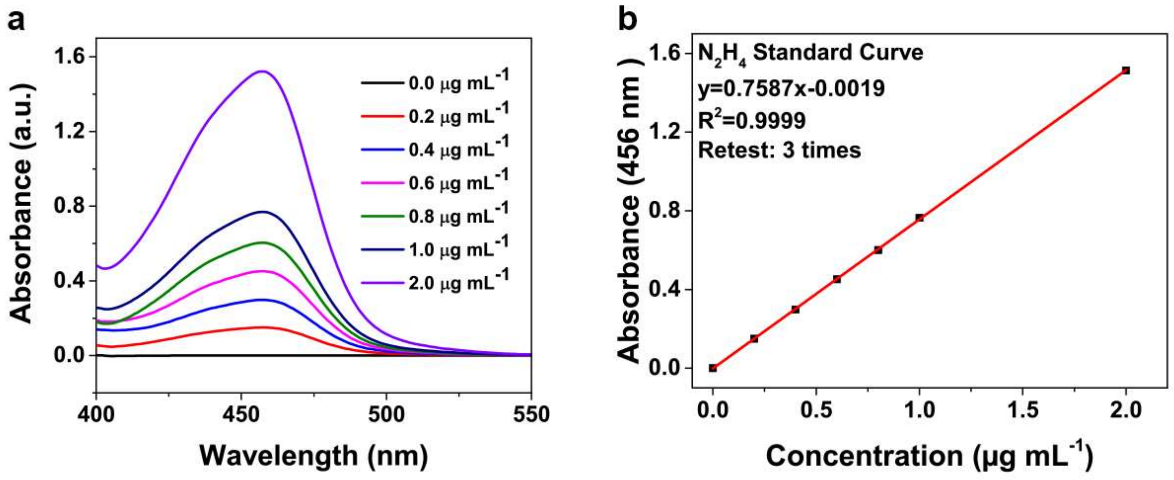

Supplementary Fig. 24 The determination of $N_2H_4 \cdot H_2O$ concentration using Watt-Chrisp method. (**a**) UV-vis spectra of a set of $N_2H_4 \cdot H_2O$ solutions with different concentrations. (**b**) The calibration curve.

Supplementary Tables

Supplementary Table 1. A summary of the ENNR performance of different catalysts reported in aqueous solution under ambient conditions and at room temperature.

**Entry**	**Potential (V)**	**NH_3 yield rate ($\mu g.\, h^{-1}.\, mg_{cat.}^{-1}$)**	**Faradic efficiency (FE, %)**	**References**
1	0.0	0.213	1.96	Acs. Sus. Chem. Eng., 2017, 5, 10986
2	0.0	7.6	56.5	Nat. Commun., 2019, 10, 341
3	-0.1	4.5	8.2	Nat. Commun., 2018, 9, 1795
4	-0.2	8.3	10.10	Adv. Mater. 2017, 29, 1700001
5	-0.2	23.88	0.217	J. Mater. Chem. A, 2018, 6, 3211
6	-0.2	23.21	10.16	Angew. Chem. Int. Ed. 2018, 57, 6073
7	-0.2	34.83	9.28	ACS Catal., 2019, 9, 336
8	-0.2	21.4	8.11	Adv. Mater., 2017, 29, 1606550
9	-0.2	8.09	11.59	Angew. Chem. Int. Ed. 2018, 57, 10246
10	-0.3	11.3	7.8	Adv. Mater., 2018, 30, 1803694
11	-0.3	34.0	14.6	Angew. Chem. Int. Ed. 2019, 58, 2321
12	-0.3	7.75	13.79	Small 2019, 15, 1805029
13	-0.4	20.4	9.3	J. Mater. Chem. A, 2018, 6, 24031

14	-0.4	29.28	8.34	Adv. Energy Mater. 2018, 8, 1801357
15	-0.5	29.43	0.75	J. Mater. Chem. A, 2018, 6, 12974
16	-0.5	8.6	10.04	Small Methods 2018, 1800333
17	-0.55	43.6	9.26	Nano Energy 2018, 52, 264
18	-0.7	23.32	6.7	Chem. Commun., 2018, 54, 11332
19	-0.7	16.22	1.84	Nanoscale Adv., 2019, 1, 961
20	-0.7	31.37	3.09	Angew. Chem. Int. Ed. 2019, 58, 261
21	-0.75	26.57	15.95	Nat. Commun., 2018, 9, 3485
22	-0.75	28.13	8.56	Chem. Commun., 2018, 54, 12848
23	-0.8	15.9	0.94	ChemCatChem 2018, 10, 4530
24	-0.8	17.04	4.76	Electrochimica Acta 2019, 298, 106
25	-0.9	23.8	< 1.5	ACS Catal. 2018, 8, 1186
26	-0.9	15.13	3.3	J. Mater. Chem. A, 2018, 6, 17303
**Au_4Pd_2/G**	**-0.2**	**27.1**	**12.3**	**This work**
**Au_4Pt_2/G**	**-0.1**	**23.6**	**9.7**	**This work**

Supplementary Table 2. Crystal data and structure refinement for $Au_8Pt_4(SR)_{16}$

Chemical formula	$C_{135}H_{152}Au_8Pt_4S_{16}$ (one toluene molecule involved)	
Formula weight	4643.61 g/mol	
Temperature	100(2) K	
Wavelength	0.71073 Å	
Crystal size	(0.057 x 0.063 x 0.425) mm^3	
Crystal system	monoclinic	
Space group	P 1 2(1)/n 1	
Unit cell dimensions	a = 18.0367(7) Å	α = 90°
	b = 30.0336(10)Å	β = 94.6750(10)°
	c = 24.5973(8) Å	γ = 90°
Volume	13280.2(8) $Å^3$	
Z	2	
Density (calculated)	2.323 g/cm^3	
Absorption coefficient	13.294 mm^{-1}	
F(000)	8648	
Theta range for data collection	2.641 to 28.294°.	
Index ranges	-24<=h<=23, -40<=k<=40, -32<=l<=32	
Reflections collected	128341	
Independent reflections	32870 [R(int) = 0.0471]	
Coverage of independent reflections	99.7%	
Absorption correction	Semi-empirical from equivalents	
Max. and min. transmission	0.7457 and 0.4177	
Refinement method	Full-matrix least-squares on F^2	
Refinement program	SHELXL-2014/7 (Sheldrick, 2014)	
Function minimized	Σ w(F_o^2 - F_c^2)^2	
Data / restraints / parameters	27998 / 0 / 1469	
Goodness-of-fit on F^2	1.056	
Final R indices	20135 data; I>2σ(I)	R1 = 0.0286, wR2 = 0.0600
	all data	R1 = 0.0420, wR2 = 0.0652
Weighting scheme	$w=1/[\sigma^2(F_o^2)+(0.0072P)^2+42.5271P]$, where $P=(F_o^2+2F_c^2)/3$	
Largest diff. peak and hole	1.787 and -2.081 e.$Å^{-3}$	
R.M.S. deviation from mean	0.197 e$Å^{-3}$	

Supplementary Table 3. The ammonia yield rate normalized by the mass (Au_4Pt_2/G).

Potential (V vs. RHE)	Absorption peak (a.u.)	C(NH_4^+) in electrolyte (µg/ml)	Volume of electrolyte (ml)	Mass of NH_4^+ (µg)	Mass of Au_4Pt_2 cluster (mg)	**Mass normalized yield rate (µg.mg^{-1}.h^{-1})**
-0.1	0.0270	0.0669	30	2.0087	0.085	23.63
-0.2	0.0194	0.0480	30	1.4398	0.085	16.94
-0.3	0.0165	0.0408	30	1.2239	0.085	14.39
-0.4	0.0131	0.0323	30	0.9683	0.085	11.39

Supplementary Table 4. The ammonia yield rate normalized by the geometric area (Au_4Pt_2/G).

Potential (V vs. RHE)	Mass of NH_4^+ (μg)	Geometric area (cm^{-2})	Time (hour)	**Area normalized yield rate ($μg.h^{-1}.cm^{-2}$)**
-0.1	2.00	4	1	0.50
-0.2	1.44	4	1	0.36
-0.3	1.22	4	1	0.30
-0.4	0.97	4	1	0.24

Supplementary Table 5. Crystal data and structure refinement for $Au_8Pd_4(SR)_{16}$

Chemical formula	$C_{135}H_{152}Au_8Pd_4S_{16}$ (one toluene molecule involved)	
Formula weight	4289.86 g/mol	
Temperature	100(2) K	
Wavelength	0.71073 Å	
Crystal size	(0.238 x 0.263 x 0.542) mm^3	
Crystal system	monoclinic	
Space group	P 1 2(1)/n 1	
Unit cell dimensions	a = 18.0520(7) Å	α = 90°
	b = 30.0619(12) Å	β = 94.697(2)°
	c = 24.5925(9) Å	γ = 90°
Volume	13301.0(9) $Å^3$	
Z	4	
Density (calculated)	2.142 g/cm^3	
Absorption coefficient	9.611 mm^{-1}	
F(000)	8140	
Theta range for data collection	2.14 to 28.70°	
Index ranges	-24<=h<=24, -40<=k<=40, -33<=l<=33	
Reflections collected	176069	
Independent reflections	34238 [R(int) = 0.0774]	
Coverage of independent reflections	99.6%	
Absorption correction	Multi-Scan	
Max. and min. transmission	0.2080 and 0.0780	
Refinement method	Full-matrix least-squares on F^2	
Refinement program	SHELXL-2014/7 (Sheldrick, 2014)	
Function minimized	Σ w(F_o^2 - F_c^2)^2	
Data / restraints / parameters	34238 / 12 / 1470	
Goodness-of-fit on F^2	1.040	
Final R indices	26648 data; I>2σ(I)	R1 = 0.0490, wR2 = 0.1172
	all data	R1 = 0.0717, wR2 = 0.1302
Weighting scheme	$w=1/[\sigma^2(F_o^2)+(0.0664P)^2+137.7858P]$ where $P=(F_o^2+2F_c^2)/3$	
Largest diff. peak and hole	5.069 and -5.408 $eÅ^{-3}$	
R.M.S. deviation from mean	0.379 $eÅ^{-3}$	
