# Supplementary material for: Atomically-precise dopant-controlled single cluster catalysis for electrochemical nitrogen reduction
Source: Nat Commun. 2020 Sep 1;11:4389. doi: 10.1038/s41467-020-18080-w (PMC7463028; doi:10.1038/s41467-020-18080-w)
Supplement: Supplementary file 3 — Description of Additional Supplementary Files [file 41467_2020_18080_MOESM3_ESM.pdf]

Description of Additional Supplementary Files

File Name: Supplementary Data 1
Description: CIFcheck document for $Au_4Pt_2(SR)_8$ cluster

File Name: Supplementary Data 2
Description: CIFcheck document for $Au_4Pd_2(SR)_8$ cluster
