# Supplementary material for: Atomically-precise dopant-controlled single cluster catalysis for electrochemical nitrogen reduction
Source: Nat Commun. 2020 Sep 1;11:4389. doi: 10.1038/s41467-020-18080-w (PMC7463028; doi:10.1038/s41467-020-18080-w)

checkCIF/PLATON report

Structure factors have been supplied for datablock(s) J081

THIS REPORT IS FOR GUIDANCE ONLY. IF USED AS PART OF A REVIEW PROCEDURE FOR PUBLICATION, IT SHOULD NOT REPLACE THE EXPERTISE OF AN EXPERIENCED CRYSTALLOGRAPHIC REFEREE.

No syntax errors found. CIF dictionary Interpreting this report

Datablock: J081

Bond precision: C-C = 0.0079 A Wavelength=0.71073

Cell:	a=18.0367(7)	b=30.0336(10)	c=24.5973(8)
	alpha=90	beta=94.675(1)	gamma=90
Temperature:	100 K		

	Calculated	Reported
Volume	13280.2(8)	13280.2(8)
Space group	P 21/n	P 21/n
Hall group	-P 2yn	-P 2yn
Moiety formula	C128 H144 Au8 Pt4 S16, C7 H8	?
Sum formula	C135 H152 Au8 Pt4 S16	C270 H304 Au16 Pt8 S32
Mr	4643.58	9287.22
Dx,g cm-3	2.322	2.323
Z	4	2
Mu (mm-1)	13.294	13.294
F000	8648.0	8648.0
F000'	8574.04	
h,k,lmax	24,40,32	24,40,32
Nref	32979	32870
Tmin,Tmax	0.330,0.528	0.418,0.746
Tmin'	0.001	

Correction method= # Reported T Limits: Tmin=0.418 Tmax=0.746
AbsCorr = MULTI-SCAN

Data completeness= 0.997 Theta(max)= 28.294

R(reflections)= 0.0286(27127) wR2(reflections)= 0.0652(32870)

S = 1.056 Npar= 1469

The following ALERTS were generated. Each ALERT has the format
test-name_ALERT_alert-type_alert-level.
Click on the hyperlinks for more details of the test.

Alert level A

```
PLAT934_ALERT_3_A Number of (Iobs-Icalc)/Sigma(W) > 10 Outliers ..        14 Check
```

Alert level B

```
PLAT910_ALERT_3_B Missing # of FCF Reflection(s) Below Theta(Min).        28 Note
PLAT919_ALERT_3_B Reflection # Likely Affected by the Beamstop ...         1 Check
```

Alert level C

```
PLAT220_ALERT_2_C NonSolvent Resd 1  C   Ueq(max) / Ueq(min) Range       3.6 Ratio
PLAT250_ALERT_2_C Large U3/U1 Ratio for Average U(i,j) Tensor ....       2.1 Note
PLAT911_ALERT_3_C Missing FCF Refl Between Thmin & STh/L=    0.600        48 Report
PLAT971_ALERT_2_C Check Calcd Resid. Dens.  0.83A   From Au3            1.57 eA-3
PLAT971_ALERT_2_C Check Calcd Resid. Dens.  1.31A   From Au8            1.56 eA-3
PLAT971_ALERT_2_C Check Calcd Resid. Dens.  0.87A   From Au2            1.56 eA-3
PLAT971_ALERT_2_C Check Calcd Resid. Dens.  1.15A   From Au1            1.54 eA-3
PLAT971_ALERT_2_C Check Calcd Resid. Dens.  0.95A   From Au8            1.53 eA-3
PLAT972_ALERT_2_C Check Calcd Resid. Dens.  0.78A   From Au7           -2.10 eA-3
PLAT972_ALERT_2_C Check Calcd Resid. Dens.  0.65A   From Au2           -1.92 eA-3
PLAT972_ALERT_2_C Check Calcd Resid. Dens.  0.85A   From Au3           -1.90 eA-3
PLAT972_ALERT_2_C Check Calcd Resid. Dens.  0.79A   From Pt3           -1.90 eA-3
PLAT972_ALERT_2_C Check Calcd Resid. Dens.  0.85A   From Pt2           -1.88 eA-3
PLAT972_ALERT_2_C Check Calcd Resid. Dens.  0.61A   From Pt1           -1.88 eA-3
PLAT972_ALERT_2_C Check Calcd Resid. Dens.  0.86A   From Au7           -1.87 eA-3
PLAT972_ALERT_2_C Check Calcd Resid. Dens.  0.63A   From Au5           -1.85 eA-3
PLAT972_ALERT_2_C Check Calcd Resid. Dens.  0.81A   From Pt4           -1.84 eA-3
PLAT972_ALERT_2_C Check Calcd Resid. Dens.  0.66A   From Pt2           -1.82 eA-3
PLAT972_ALERT_2_C Check Calcd Resid. Dens.  0.81A   From Au6           -1.82 eA-3
PLAT972_ALERT_2_C Check Calcd Resid. Dens.  0.87A   From Pt2           -1.82 eA-3
PLAT972_ALERT_2_C Check Calcd Resid. Dens.  0.87A   From Pt1           -1.80 eA-3
PLAT972_ALERT_2_C Check Calcd Resid. Dens.  0.86A   From Au3           -1.80 eA-3
PLAT972_ALERT_2_C Check Calcd Resid. Dens.  0.67A   From Au8           -1.75 eA-3
PLAT972_ALERT_2_C Check Calcd Resid. Dens.  0.73A   From Au5           -1.74 eA-3
PLAT972_ALERT_2_C Check Calcd Resid. Dens.  0.75A   From Au6           -1.73 eA-3
PLAT972_ALERT_2_C Check Calcd Resid. Dens.  0.71A   From Pt3           -1.70 eA-3
PLAT972_ALERT_2_C Check Calcd Resid. Dens.  0.77A   From Au5           -1.68 eA-3
PLAT972_ALERT_2_C Check Calcd Resid. Dens.  0.81A   From Pt1           -1.68 eA-3
PLAT972_ALERT_2_C Check Calcd Resid. Dens.  0.91A   From Au8           -1.65 eA-3
PLAT972_ALERT_2_C Check Calcd Resid. Dens.  0.79A   From Au1           -1.63 eA-3
PLAT972_ALERT_2_C Check Calcd Resid. Dens.  0.85A   From Au8           -1.62 eA-3
PLAT972_ALERT_2_C Check Calcd Resid. Dens.  1.39A   From Au5           -1.61 eA-3
PLAT972_ALERT_2_C Check Calcd Resid. Dens.  0.81A   From Au1           -1.61 eA-3
PLAT977_ALERT_2_C Check Negative Difference Density on H5              -0.35 eA-3
PLAT977_ALERT_2_C Check Negative Difference Density on H7SC            -0.34 eA-3
PLAT977_ALERT_2_C Check Negative Difference Density on H10B            -0.32 eA-3
PLAT977_ALERT_2_C Check Negative Difference Density on H10D            -0.36 eA-3
PLAT977_ALERT_2_C Check Negative Difference Density on H16             -0.39 eA-3
PLAT977_ALERT_2_C Check Negative Difference Density on H21             -0.36 eA-3
PLAT977_ALERT_2_C Check Negative Difference Density on H26A            -0.41 eA-3
PLAT977_ALERT_2_C Check Negative Difference Density on H30             -0.51 eA-3
PLAT977_ALERT_2_C Check Negative Difference Density on H38             -0.36 eA-3
PLAT977_ALERT_2_C Check Negative Difference Density on H40             -0.53 eA-3
PLAT977_ALERT_2_C Check Negative Difference Density on H54             -0.31 eA-3
PLAT977_ALERT_2_C Check Negative Difference Density on H60             -0.43 eA-3
PLAT977_ALERT_2_C Check Negative Difference Density on H74B            -0.33 eA-3
```

```
PLAT977_ALERT_2_C Check Negative Difference Density on H89A            -0.60 eA-3
PLAT977_ALERT_2_C Check Negative Difference Density on H97A            -0.36 eA-3
PLAT977_ALERT_2_C Check Negative Difference Density on H98B            -0.50 eA-3
PLAT977_ALERT_2_C Check Negative Difference Density on H126            -0.38 eA-3
```

Alert level G

```
PLAT004_ALERT_5_G Polymeric Structure Found with Maximum Dimension          1 Info
PLAT045_ALERT_1_G Calculated and Reported Z Differ by a Factor ...       2.00 Check
PLAT083_ALERT_2_G SHELXL Second Parameter in WGHT  Unusually Large      11.33 Why ?
PLAT720_ALERT_4_G Number of Unusual/Non-Standard Labels ..........          6 Note
PLAT794_ALERT_5_G Tentative Bond Valency for Au2       (III)     .       2.55 Info
PLAT794_ALERT_5_G Tentative Bond Valency for Au3       (III)     .       2.54 Info
PLAT794_ALERT_5_G Tentative Bond Valency for Au6       (III)     .       2.60 Info
PLAT794_ALERT_5_G Tentative Bond Valency for Au7       (III)     .       2.49 Info
PLAT794_ALERT_5_G Tentative Bond Valency for Pt1       (II)      .       2.50 Info
PLAT794_ALERT_5_G Tentative Bond Valency for Pt2       (II)      .       2.52 Info
PLAT794_ALERT_5_G Tentative Bond Valency for Pt3       (II)      .       2.51 Info
PLAT794_ALERT_5_G Tentative Bond Valency for Pt4       (II)      .       2.51 Info
PLAT883_ALERT_1_G No Info/Value for _atom_sites_solution_primary .     Please Do !
PLAT912_ALERT_4_G Missing # of FCF Reflections Above STh/L=  0.600         34 Note
PLAT913_ALERT_3_G Missing # of Very Strong Reflections in FCF ....          3 Note
PLAT933_ALERT_2_G Number of OMIT Records in Embedded .res File ...         23 Note
PLAT941_ALERT_3_G Average HKL Measurement Multiplicity ...........        3.9 Low
PLAT965_ALERT_2_G The SHELXL WEIGHT Optimisation has not Converged     Please Check
PLAT978_ALERT_2_G Number C-C Bonds with Positive Residual Density.          0 Info
```

```
  1 ALERT level A = Most likely a serious problem - resolve or explain
  2 ALERT level B = A potentially serious problem, consider carefully
 50 ALERT level C = Check. Ensure it is not caused by an omission or oversight
 19 ALERT level G = General information/check it is not something unexpected

  2 ALERT type 1 CIF construction/syntax error, inconsistent or missing data
 53 ALERT type 2 Indicator that the structure model may be wrong or deficient
  6 ALERT type 3 Indicator that the structure quality may be low
  2 ALERT type 4 Improvement, methodology, query or suggestion
  9 ALERT type 5 Informative message, check
```

It is advisable to attempt to resolve as many as possible of the alerts in all categories. Often the minor alerts point to easily fixed oversights, errors and omissions in your CIF or refinement strategy, so attention to these fine details can be worthwhile. In order to resolve some of the more serious problems it may be necessary to carry out additional measurements or structure refinements. However, the purpose of your study may justify the reported deviations and the more serious of these should normally be commented upon in the discussion or experimental section of a paper or in the "special_details" fields of the CIF. checkCIF was carefully designed to identify outliers and unusual parameters, but every test has its limitations and alerts that are not important in a particular case may appear. Conversely, the absence of alerts does not guarantee there are no aspects of the results needing attention. It is up to the individual to critically assess their own results and, if necessary, seek expert advice.

Publication of your CIF in IUCr journals

A basic structural check has been run on your CIF. These basic checks will be run on all CIFs submitted for publication in IUCr journals (*Acta Crystallographica*, *Journal of Applied Crystallography*, *Journal of Synchrotron Radiation*); however, if you intend to submit to *Acta Crystallographica Section C* or *E* or *IUCrData*, you should make sure that full publication checks are run on the final version of your CIF prior to submission.

Publication of your CIF in other journals

Please refer to the *Notes for Authors* of the relevant journal for any special instructions relating to CIF submission.

Validation response form

Please find below a validation response form (VRF) that can be filled in and pasted into your CIF.

```
# start Validation Reply Form
_vrf_PLAT934_J081
;
PROBLEM: Number of (Iobs-Icalc)/Sigma(W) > 10 Outliers ..         14 Check
RESPONSE: ...
;
_vrf_PLAT220_J081
;
PROBLEM: NonSolvent Resd 1  C   Ueq(max) / Ueq(min) Range        3.6 Ratio
RESPONSE: ...
;
_vrf_PLAT250_J081
;
PROBLEM: Large U3/U1 Ratio for Average U(i,j) Tensor ....        2.1 Note
RESPONSE: ...
;
_vrf_PLAT911_J081
;
PROBLEM: Missing FCF Refl Between Thmin & STh/L=    0.600         48 Report
RESPONSE: ...
;
_vrf_PLAT971_J081
;
PROBLEM: Check Calcd Resid. Dens.  0.83A   From Au3             1.57 eA-3
RESPONSE: ...
;
_vrf_PLAT972_J081
```

```
;
PROBLEM: Check Calcd Resid. Dens.  0.78A   From Au7            -2.10 eA-3
RESPONSE: ...
;
_vrf_PLAT977_J081
;
PROBLEM: Check Negative Difference Density on H5               -0.35 eA-3
RESPONSE: ...
;
# end Validation Reply Form
```

PLATON version of 04/06/2020; check.def file version of 02/06/2020

Datablock J081 - ellipsoid plot

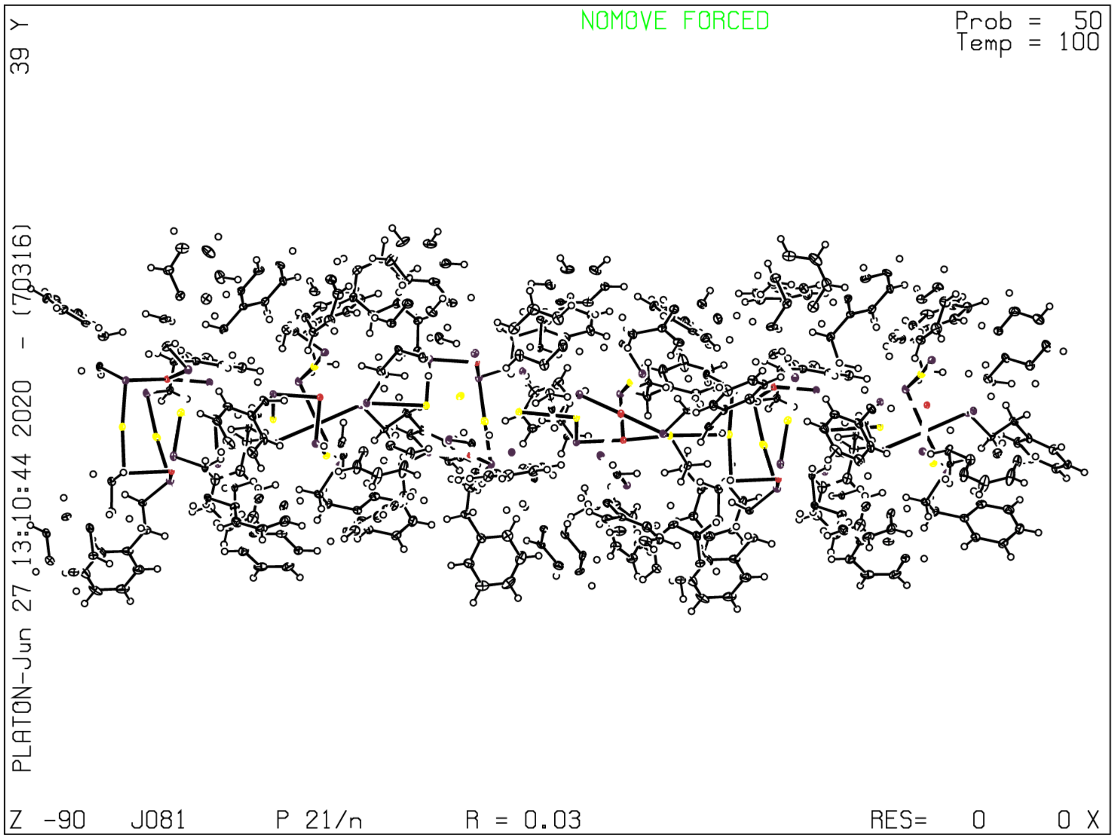

Supplement: Supplementary file 4 — Supplementary Data 1 [file 41467_2020_18080_MOESM4_ESM.pdf]
