# Supplementary material for: Atomically-precise dopant-controlled single cluster catalysis for electrochemical nitrogen reduction
Source: Nat Commun. 2020 Sep 1;11:4389. doi: 10.1038/s41467-020-18080-w (PMC7463028; doi:10.1038/s41467-020-18080-w)

checkCIF/PLATON report

Structure factors have been supplied for datablock(s) I067

THIS REPORT IS FOR GUIDANCE ONLY. IF USED AS PART OF A REVIEW PROCEDURE FOR PUBLICATION, IT SHOULD NOT REPLACE THE EXPERTISE OF AN EXPERIENCED CRYSTALLOGRAPHIC REFEREE.

No syntax errors found.   CIF dictionary   Interpreting this report

Datablock: I067

```
Bond precision:  C-C = 0.0100 A          Wavelength=0.71073

Cell:            a=18.0464(6)     b=30.0220(11)      c=24.5955(7)
                 alpha=90         beta=94.672(1)     gamma=90
Temperature:     100 K
```

	Calculated	Reported
Volume	13281.3(8)	13281.3(8)
Space group	P 21/n	P 21/n
Hall group	-P 2yn	-P 2yn
Moiety formula	C128 H144 Au8 Pd4 S16, C7 H8	?
Sum formula	C135 H152 Au8 Pd4 S16	C135 H152 Au8 Pd4 S16
Mr	4288.87	4288.85
Dx,g cm-3	2.145	2.145
Z	4	4
Mu (mm-1)	9.626	9.626
F000	8136.0	8136.0
F000’	8069.35	
h,k,lmax	24,40,32	24,40,32
Nref	32950	32843
Tmin,Tmax	0.153,0.412	0.483,0.746
Tmin’	0.070	

```
Correction method= # Reported T Limits: Tmin=0.483 Tmax=0.746
AbsCorr = MULTI-SCAN

Data completeness= 0.997          Theta(max)= 28.282

R(reflections)= 0.0341( 25926)    wR2(reflections)= 0.0822( 32843)

S = 1.077                  Npar= 1469
```

The following ALERTS were generated. Each ALERT has the format
test-name_ALERT_alert-type_alert-level.
Click on the hyperlinks for more details of the test.

Alert level B

```
PLAT910_ALERT_3_B Missing # of FCF Reflection(s) Below Theta(Min).         14 Note
PLAT919_ALERT_3_B Reflection # Likely Affected by the Beamstop ...          4 Check
PLAT934_ALERT_3_B Number of (Iobs-Icalc)/Sigma(W) > 10 Outliers ..          9 Check
```

Alert level C

```
PLAT213_ALERT_2_C Atom C7              has ADP max/min Ratio .....        3.1 prolat
PLAT213_ALERT_2_C Atom C19             has ADP max/min Ratio .....        3.2 oblate
PLAT220_ALERT_2_C NonSolvent Resd 1  C   Ueq(max) / Ueq(min) Range        3.5 Ratio
PLAT250_ALERT_2_C Large U3/U1 Ratio for Average U(i,j) Tensor ....        2.3 Note
PLAT342_ALERT_3_C Low Bond Precision on  C-C Bonds ...............       0.01 Ang.
PLAT911_ALERT_3_C Missing FCF Refl Between Thmin & STh/L=    0.600         14 Report
PLAT913_ALERT_3_C Missing # of Very Strong Reflections in FCF ....          4 Note
PLAT918_ALERT_3_C Reflection(s) with I(obs) much Smaller I(calc) .          2 Check
PLAT939_ALERT_3_C Large Value of Not (SHELXL) Weight Optimized S .      14.63 Check
PLAT971_ALERT_2_C Check Calcd Resid. Dens.  0.83A   From Au4             2.07 eA-3
PLAT971_ALERT_2_C Check Calcd Resid. Dens.  0.98A   From Au4             1.69 eA-3
PLAT972_ALERT_2_C Check Calcd Resid. Dens.  0.76A   From Au8            -1.70 eA-3
PLAT973_ALERT_2_C Check Calcd Positive Resid. Density on       Au4       1.31 eA-3
PLAT977_ALERT_2_C Check Negative Difference Density on H1SC             -0.38 eA-3
PLAT977_ALERT_2_C Check Negative Difference Density on H3S              -0.45 eA-3
PLAT977_ALERT_2_C Check Negative Difference Density on H7               -0.34 eA-3
PLAT977_ALERT_2_C Check Negative Difference Density on H7S              -0.41 eA-3
PLAT977_ALERT_2_C Check Negative Difference Density on H13              -0.36 eA-3
PLAT977_ALERT_2_C Check Negative Difference Density on H18A             -0.35 eA-3
PLAT977_ALERT_2_C Check Negative Difference Density on H18B             -0.49 eA-3
PLAT977_ALERT_2_C Check Negative Difference Density on H24              -0.35 eA-3
PLAT977_ALERT_2_C Check Negative Difference Density on H33A             -0.42 eA-3
PLAT977_ALERT_2_C Check Negative Difference Density on H39              -0.33 eA-3
PLAT977_ALERT_2_C Check Negative Difference Density on H41B             -0.54 eA-3
PLAT977_ALERT_2_C Check Negative Difference Density on H56              -0.46 eA-3
PLAT977_ALERT_2_C Check Negative Difference Density on H58B             -0.52 eA-3
PLAT977_ALERT_2_C Check Negative Difference Density on H60              -0.59 eA-3
PLAT977_ALERT_2_C Check Negative Difference Density on H61              -0.41 eA-3
PLAT977_ALERT_2_C Check Negative Difference Density on H69              -0.37 eA-3
PLAT977_ALERT_2_C Check Negative Difference Density on H76              -0.55 eA-3
PLAT977_ALERT_2_C Check Negative Difference Density on H89B             -0.36 eA-3
PLAT977_ALERT_2_C Check Negative Difference Density on H93              -0.45 eA-3
PLAT977_ALERT_2_C Check Negative Difference Density on H117             -0.47 eA-3
PLAT977_ALERT_2_C Check Negative Difference Density on H118             -0.40 eA-3
PLAT977_ALERT_2_C Check Negative Difference Density on H126             -0.59 eA-3
PLAT977_ALERT_2_C Check Negative Difference Density on H127             -0.50 eA-3
```

Alert level G

```
PLAT003_ALERT_2_G Number of Uiso or Uij Restrained non-H Atoms ...          1 Report
PLAT004_ALERT_5_G Polymeric Structure Found with Maximum Dimension          1 Info
PLAT083_ALERT_2_G SHELXL Second Parameter in WGHT  Unusually Large      16.01 Why ?
PLAT186_ALERT_4_G The CIF-Embedded .res File Contains ISOR Records          1 Report
PLAT720_ALERT_4_G Number of Unusual/Non-Standard Labels ..........          3 Note
PLAT764_ALERT_4_G Overcomplete CIF Bond List Detected (Rep/Expd) .       1.10 Ratio
PLAT794_ALERT_5_G Tentative Bond Valency for Au1       (III)     .       2.62 Info
PLAT794_ALERT_5_G Tentative Bond Valency for Au4       (III)     .       2.51 Info
PLAT794_ALERT_5_G Tentative Bond Valency for Au6       (III)     .       2.54 Info
PLAT794_ALERT_5_G Tentative Bond Valency for Au8       (III)     .       2.56 Info
```

```
PLAT794_ALERT_5_G Tentative Bond Valency for Pd1       (II)      .       2.01 Info
PLAT794_ALERT_5_G Tentative Bond Valency for Pd2       (II)      .       2.02 Info
PLAT794_ALERT_5_G Tentative Bond Valency for Pd3       (II)      .       2.03 Info
PLAT794_ALERT_5_G Tentative Bond Valency for Pd4       (II)      .       2.01 Info
PLAT860_ALERT_3_G Number of Least-Squares Restraints .............          6 Note
PLAT883_ALERT_1_G No Info/Value for _atom_sites_solution_primary .     Please Do !
PLAT912_ALERT_4_G Missing # of FCF Reflections Above STh/L=  0.600         79 Note
PLAT978_ALERT_2_G Number C-C Bonds with Positive Residual Density.          0 Info
```

```
   0 ALERT level A = Most likely a serious problem - resolve or explain
   3 ALERT level B = A potentially serious problem, consider carefully
  36 ALERT level C = Check. Ensure it is not caused by an omission or oversight
  18 ALERT level G = General information/check it is not something unexpected

   1 ALERT type 1 CIF construction/syntax error, inconsistent or missing data
  34 ALERT type 2 Indicator that the structure model may be wrong or deficient
   9 ALERT type 3 Indicator that the structure quality may be low
   4 ALERT type 4 Improvement, methodology, query or suggestion
   9 ALERT type 5 Informative message, check
```

Validation response form

Please find below a validation response form (VRF) that can be filled in and pasted into your CIF.

```
# start Validation Reply Form
_vrf_PLAT213_I067
;
PROBLEM: Atom C7              has ADP max/min Ratio .....        3.1 prolat
RESPONSE: ...
;
_vrf_PLAT220_I067
;
PROBLEM: NonSolvent Resd 1  C   Ueq(max) / Ueq(min) Range        3.5 Ratio
RESPONSE: ...
;
_vrf_PLAT250_I067
;
PROBLEM: Large U3/U1 Ratio for Average U(i,j) Tensor ....        2.3 Note
RESPONSE: ...
;
_vrf_PLAT342_I067
;
PROBLEM: Low Bond Precision on  C-C Bonds ..............       0.01 Ang.
RESPONSE: ...
;
_vrf_PLAT911_I067
;
PROBLEM: Missing FCF Refl Between Thmin & STh/L=    0.600         14 Report
RESPONSE: ...
;
_vrf_PLAT913_I067
;
PROBLEM: Missing # of Very Strong Reflections in FCF ....          4 Note
RESPONSE: ...
;
_vrf_PLAT918_I067
;
PROBLEM: Reflection(s) with I(obs) much Smaller I(calc) .          2 Check
RESPONSE: ...
```

```
;
_vrf_PLAT939_I067
;
PROBLEM: Large Value of Not (SHELXL) Weight Optimized S .      14.63 Check
RESPONSE: ...
;
_vrf_PLAT971_I067
;
PROBLEM: Check Calcd Resid. Dens.  0.83A   From Au4             2.07 eA-3
RESPONSE: ...
;
_vrf_PLAT972_I067
;
PROBLEM: Check Calcd Resid. Dens.  0.76A   From Au8            -1.70 eA-3
RESPONSE: ...
;
_vrf_PLAT973_I067
;
PROBLEM: Check Calcd Positive Resid. Density on       Au4       1.31 eA-3
RESPONSE: ...
;
_vrf_PLAT977_I067
;
PROBLEM: Check Negative Difference Density on H1SC             -0.38 eA-3
RESPONSE: ...
;
# end Validation Reply Form
```

It is advisable to attempt to resolve as many as possible of the alerts in all categories. Often the minor alerts point to easily fixed oversights, errors and omissions in your CIF or refinement strategy, so attention to these fine details can be worthwhile. In order to resolve some of the more serious problems it may be necessary to carry out additional measurements or structure refinements. However, the purpose of your study may justify the reported deviations and the more serious of these should normally be commented upon in the discussion or experimental section of a paper or in the "special_details" fields of the CIF. checkCIF was carefully designed to identify outliers and unusual parameters, but every test has its limitations and alerts that are not important in a particular case may appear. Conversely, the absence of alerts does not guarantee there are no aspects of the results needing attention. It is up to the individual to critically assess their own results and, if necessary, seek expert advice.

Publication of your CIF in IUCr journals

A basic structural check has been run on your CIF. These basic checks will be run on all CIFs submitted for publication in IUCr journals (*Acta Crystallographica*, *Journal of Applied Crystallography*, *Journal of Synchrotron Radiation*); however, if you intend to submit to *Acta Crystallographica Section C* or *E* or *IUCrData*, you should make sure that full publication checks are run on the final version of your CIF prior to submission.

Publication of your CIF in other journals

Please refer to the *Notes for Authors* of the relevant journal for any special instructions relating to CIF submission.

PLATON version of 04/06/2020; check.def file version of 02/06/2020

Datablock I067 - ellipsoid plot

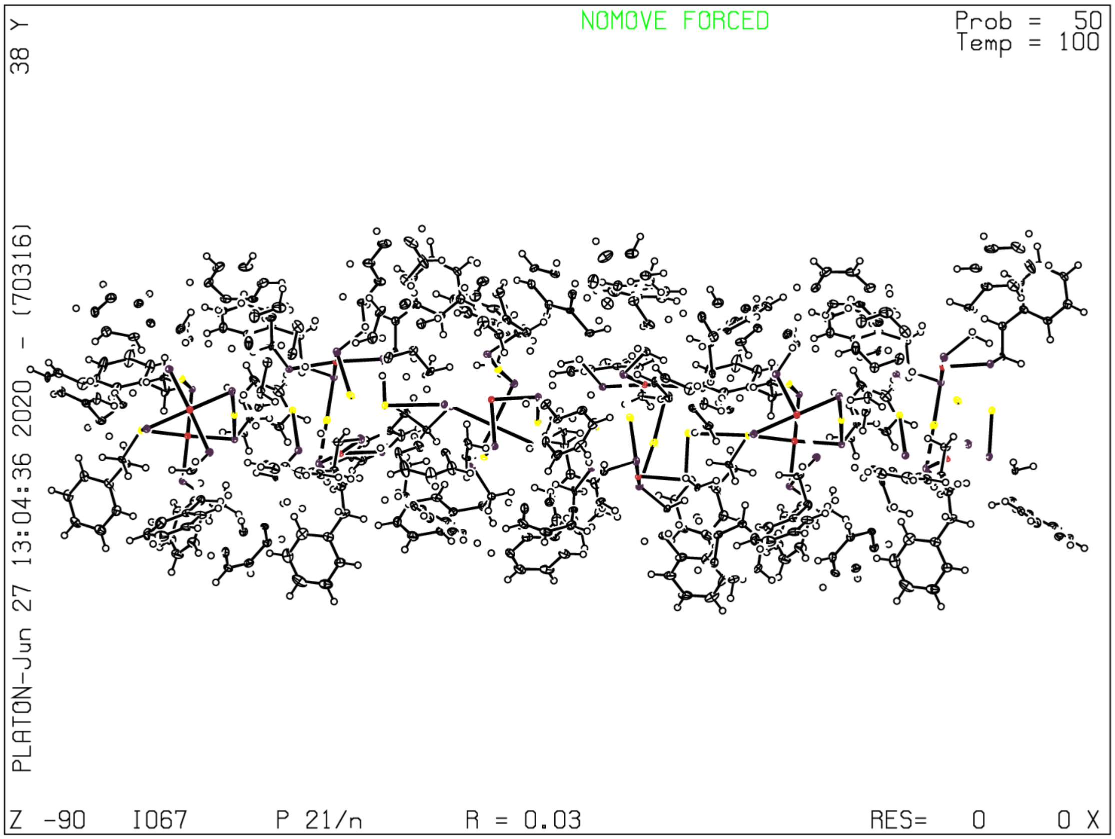

Supplement: Supplementary file 5 — Supplementary Data 2 [file 41467_2020_18080_MOESM5_ESM.pdf]
